# A *cis*-Acting Element Present within the *gag* Open Reading Frame Negatively Impacts on the Activity of the HIV-1 IRES

**DOI:** 10.1371/journal.pone.0056962

**Published:** 2013-02-25

**Authors:** Fernando Valiente-Echeverría, Maricarmen Vallejos, Anne Monette, Karla Pino, Alejandro Letelier, J. Pablo Huidobro-Toro, Andrew J. Mouland, Marcelo López-Lastra

**Affiliations:** 1 Laboratorio de Virología Molecular, Instituto Milenio de Inmunología e Inmunoterapia, Centro de Investigaciones Médicas, Escuela de Medicina, Pontificia Universidad Católica de Chile, Santiago, Chile; 2 HIV-1 Trafficking Laboratory, Lady Davis Institute at the Jewish General Hospital, Montréal, Québec, Canada; 3 Department of Experimental Medicine, McGill University, Montreal, Quebec, Canada; 4 Department of Microbiology and Immunology, McGill University, Montreal, Quebec, Canada; 5 Departamento de Fisiología, Centro de Envejecimiento y Regeneración, CARE, Facultad de Ciencias Biológicas, Pontificia Universidad Católica de Chile, Santiago, Chile; University of British Columbia, Canada

## Abstract

Translation initiation from the human immunodeficiency virus type-1 (HIV-1) mRNA can occur through a cap or an IRES dependent mechanism. Cap-dependent translation initiation of the HIV-1 mRNA can be inhibited by the instability element (INS)-1, a *cis*-acting regulatory element present within the *gag* open reading frame (ORF). In this study we evaluated the impact of the INS-1 on HIV-1 IRES-mediated translation initiation. Using heterologous bicistronic mRNAs, we show that the INS-1 negatively impact on HIV-1 IRES-driven translation in *in vitro* and in cell-based experiments. Additionally, our results show that the inhibitory effect of the INS-1 is not general to all IRESes since it does not hinder translation driven by the HCV IRES. The inhibition by the INS-1 was partially rescued in cells by the overexpression of the viral Rev protein or hnRNPA1.

## Introduction

In eukaryotes, the expression of proteins is frequently subject to regulation at the level of the initiation of mRNA translation [1], [2]. Translation initiation is a stepwise process by which the 40S ribosomal subunit is recruited to the mRNA, and scans it in a 5′-3′direction until the first initiation codon (AUG) is encountered. Recognition of the initiation codon by the migrating initiation complex leads to 80S ribosome assembly. The primary mechanism of initiation of protein synthesis involves the recognition of the 5′ cap structure (m^7^GpppN, where N is any nucleotide) on the mRNAs by eukaryotic translation initiation factors (eIFs) that deliver the 40S ribosomal subunit [1], [2]. Alternatively, an RNA structure termed the internal ribosome entry site (IRES) can drive 40S ribosomal subunit recruitment and positioning on the mRNA independently from the 5′cap structure [3].

Translation initiation from the capped and polyadenylated unspliced mRNA of the human immunodeficiency virus type 1 (HIV-1) can occur through a canonical cap-dependent or by an alternative IRES-mediated mechanism (reviewed in [4], [5]). The unspliced HIV-1 mRNA harbors two IRESes, the first of which is in the 5′UTR (here referred to as the HIV-1 IRES) [6], [7], and the second of which is within the *gag* open reading frame (the HIV-1 *gag* IRES) [8]. This observed redundancy in the possible mechanisms used to initiate translation of the unspliced HIV-1 mRNA, cap- or IRES-dependent, is conserved among primate lentiviruses suggesting that translation initiation of the unspliced mRNA is a key step during the viral life cycle [4].

Cap-independent initiation has been proposed to allow the viral mRNA to bypass the constraints of global cellular translation repression that normally target cap-dependent translation initiation [1], [2], [9]. The HIV-1 IRES drives viral structural protein synthesis during oxidative and osmotic stress [7], [10], during G2/M of the cell cycle [6], [11] and even when eIF4G and the poly(A) binding protein (PABP), two proteins that are critical for cap-dependent translation initiation, are cleaved by the viral protease [12]–[15].

The molecular mechanisms that determine the function of the HIV-1 IRES element are not clearly understood. However, recent reports suggest that translation initiation driven by the HIV-1 IRES is to some extent modulated by cellular proteins [11], [16]–[18]. Evidence also suggests that the activity of the HIV-1 IRES is negatively influenced by *cis*-acting elements distinct from the 5′cap structure or the 3′poly(A) tail [7]. Brasey et al. (2003) reported that when in the context of a bicistronic mRNA, the *gag* open reading frame (ORF) negatively influences translation initiation driven from the HIV-1 5′UTR, while Gendron et al. (2011) defined another region upstream of the primer binding site (PBS), the IRES negative element (IRENE), that also impacts on activity of the HIV-1 IRES. In this study we further explore the possibility that an RNA region downstream of the Gag initiation codon acts in *cis*- to modulate the translation of the full-length HIV-1 mRNA. In fact, several *cis*-acting RNA elements, distinct from the 5′cap structure, the 3′poly(A) tail, and IRENE, are known to regulate HIV-1 Gag protein expression. These *cis*-acting elements include amongst others, the *cis*-acting repressive sequence or inhibitory sequences (CRS/INS), from here on referred to generically as the INS elements [19]–[24]. The INS elements, which are scattered throughout the *gag*, *pol*, and *env* regions of HIV-1 mRNA [19]–[24], restrict expression of HIV-1 structural proteins by yet undefined mechanisms. A consensus sequence has not been defined for these elements, but in general INSs are characterized as AU-rich elements (AREs) [23], [24]. One such element, known as the INS-1, found within the Matrix (p17) Gag coding region is reported to function as an inhibitor of cap-dependent translation initiation [19], [24]. For example, when inserted in the context of a heterologous non-viral mRNA the INS-1 inhibits protein synthesis [19], [21], [24]. This observation indicates that the inhibition of cap-dependent translation initiation by the INS element is not restricted to the context of the HIV-1 mRNA, suggesting a more general mechanism of translational control. While the molecular mechanism by which INS-1 restricts cap-dependent translation initiation remains undefined, several reports suggest that host proteins are involved [25]–[28]. Considering that the unspliced full-length HIV-1 mRNA can initiate translation by at least two distinct mechanisms, that is, through a cap-dependent or an IRES-dependent mechanism [6]–[8], [29]–[31], in this study we evaluated the impact of the INS-1 on HIV-1 IRES-driven translation initiation. Our results show that the INS-1 negatively impacts on IRES-dependent translation initiation both *in vitro* and in cells-based experiments. The INS-1 does not hinder translation driven by the HCV IRES, suggesting that the inhibitory activity of the INS-1 does not equally impact all IRES elements. The inhibition on HIV-1 IRES activity by the INS-1 is partially rescued in cells by the overexpression of either the viral protein Rev or the heterogeneous nuclear ribonucleoprotein A1 (hnRNPA1). The data presented herein describe novel features of the regulation of HIV-1 gene expression as they show that HIV-1 IRES activity is modulated by cis-acting elements present within the gagORF.

## Materials and Methods

### Plasmids

The dl ΔEMCV, dl HIV-1 IRES, and dl HCV IRES plasmid have been previously described [6], [31]. The HIV-1 genomic region containing the INS-1 sequence (nucleotides 348–734) [23], [32], was recovered from pNL4.3 (GenBank AF324493) using primers 5′-*gctctaga*GCGTCGGTATTAAGCGGGG-3′ and 5′-*gctctaga*GGGTAATTTTGGCTGACCTGGC-3′. The amplicon was digested with *XbaI* (restriction site, in lower case and italics, were added with the primer by PCR) and inserted after the FLuc reporter stop codon in the dl HIV-1 IRES plasmid. In plasmid dl HIV-1 IRES/INS the INS-1 was cloned in the sense orientation, while it was cloned in the antisense orientation in plasmid dl HIV-1 IRES/SNI. The dl HCV IRES/INS was generated using a similar strategy from the previously described dl HCV IRES plasmid [31]. The HIV-1 RRE was recovered by PCR from pNL4.3 using primers 5′-*gctctaga*GAAGAGTGGTGCAGAGAG-3′ and 5′-*cgtctaga*AGGCACAGCAGTGGTGC-3′. The amplicon was digested with *XbaI* (restriction site added by PCR) and inserted after the FLuc reporter stop codon in the dl HIV-1 IRES plasmid. For the generation of dl HIV-1 IRES/INS/RRE, the INS-1 was recovered from pNL4.3 using primers 5′-*gctcgag*GCGTCGGTATTAAGCGGGG-3′ and 5′-*gatgctagc*GGGTAATTTTGGCTGACCTGGC-3′ while the sequence of the RRE was from pNL4.3 using primers 5′-*ctagctagc*GAAGAGTGGTGCAGAAGAG-3′ and 5′-*cgtctaga*AGGCACAGCAGTGGTGC-3′. The INS-1 and RRE amplicons were digested with NheI (restriction site added by PCR), ligated and later amplified by PCR using the external primers to recover the INS/RRE segment. The INS/RRE amplicon was digested with *XbaI* and inserted after the FLuc reporter stop codon in the dl HIV-1 IRES plasmid. The pRFP and pRev-R-YC plasmids were kindly provided by R. Brack-Werner (Helmholtz Zentrum München, Neuherberg, Germany) [33]. The Myc epitope-tagged hnRNP A1 expressor (pMyc-A1) was provided by Benoit Chabôt (Université de Sherbrooke, Québec, Canada) [17]. All constructs were sequenced (Macrogen, USA).

### Cell Culture

HeLa (CCL-2™) cells were grown in Dulbecco’s modified Eagle’s medium (Gibco-BRL) with 50 U/mL of penicillin-streptomycin (HyClone) and 10% fetal bovine serum (HyClone) at 37°C in a 5% CO_2_ atmosphere. Nocodazole (0.4 µg/mL; Sigma- Aldrich) was used to enrich cells in the G_2_/M phase of the cell cycle as confirmed by flow cytometry [6]. Cytoplasmic cell extracts were prepared following a previously described protocol [6], [11].

### *In vitro* Transcription

Capped and polyadenylated RNAs were synthesized using the mMESSAGE mMACHINE High Yield Capped RNA Transcription Kit (Ambion) and Poly(A) tailing kit (Ambion) according to the manufacturer’s protocol. RNA was precipitated with 2.5 M LiCl, centrifuged at 16000×g, 30 min at 4°C, washed with 70% ethanol, dried, and resuspended in 25 µl of nuclease-free water. RNA concentrations were determined spectrophotometrically (NanoDrop Technology, Wilmington, Delaware, USA) and RNA integrity was monitored by electrophoresis on denaturing agarose gels.

### *In vitro* Translation

*In vitro* transcribed RNAs (1 fmol) were translated in 35% (v/v) nuclease-treated rabbit reticulocyte lysate (RRL; Promega), supplemented with cell extracts and salts as previously described [11]. *In vitro* translation of the dl HCV IRES was performed in non-supplemented nuclease-treated rabbit reticulocyte lysate under previously described conditions [31]. Luciferase activities were measured using the DLR™ Assay System (Promega) according to the manufacturer’s instructions on a Sirius Single Tube Luminometer Lumat 9507 (Berthold Detection Systems, Germany) as previously described [11].

### DNA Transfections

HeLa cells were seeded at 2×10^5^ cell/well in 12-well plates and DNA transfections were performed at 80% confluence using Lipofectamine™ 2000 (Invitrogen™ Life Technologies) according to the manufacturer’s protocol. Cells were co-transfected with 200 ng or 1 ug (see figure legend) of bicistronic DNA vectors and 250 ng of pRev-R-YC plasmid [33], or 1 ug of pMyc-hnRNPA1 (pMyc-A1) [17]. Plasmids pRFP, expressing the red fluorescent protein, and pcDNA3.1 (Invitrogen™ Life Technologies) where used in this experiments for mock transfections, respectively. After 24 h, post-transfection culture medium was removed and cells were lysed in 1X passive lysis buffer (Promega). Protein concentrations were determined using the Bio-Rad Protein Assay (Bio-Rad). FLuc and RLuc activities were measured as described above [11].

### RNA Extraction, RT-PCR and RT-qPCR

Cells were washed with phosphate-buffered saline (PBS: 137 mM NaCl, 2.7 mM KCl, 4.3 mM Na_2_HPO_4_·7H_2_O, 1.4 mM KH_2_PO_4_, pH 7.4) at 4°C and lysed in 100 µL of RLNa buffer (10 mM Tris–HCl (pH 8.0), 10 mM NaCl, 3 mM MgCl_2_, 1 mM DTT, 0.5% NP40 and 10 U/mL of RiboLock™ RNase Inhibitor (Fermentas)) for 2 min on ice. Supernatants were mixed with 1 mL of TRIzol® Reagent (Invitrogen™ Life Technologies) and total RNA was extracted according to the manufacturer’s instructions. Total RNA was resuspended in 20 µL of nuclease-free water and treated using the DNA-free™ kit (Applied Biosystems/Ambion, Austin, TX, USA) according to the manufacturer’s protocol. The integrity of the RNA obtained was confirmed by electrophoresis on 1% agarose gels and quantified by spectrophotometry (NanoDrop Technology).

The PCR assay was conducted using primers p2anti (5′-TCTCTTCATAGCCTTATGCAGTTG-3′) and RenFw (5′-TCAAATCGTTCGTTGAGCGAGTTC-3′), 100 ng of total DNA and the Go Taq®Green Master mix (Promega), according to the manufacturer’s protocol. The RT-PCR assay was carried out with SuperScript™ III One-Step RT-PCR System using Platinum® Taq DNA polymerase (Invitrogen™ Life Technologies) according to the manufacturer’s protocol, using 1 µg of RNA and the primers described above. The RT-qPCR assay was conducted using the primers RLucS (5′-AGGTGAAGTTCGTCGTCCAACATTATC-3′) and RLucAS (5′- GAAACTTCTTGGCACCTTCAACAATAGC-3′) for RLuc amplification (193 bp); or FLucS (5′- ACTTCGAAATGTCCGTTCGG-3′) and FLucAS (5′- GCAACTCCGATAAATAACGCG-3′) for FLuc amplification (135 bp), using the Brilliant® II SYBR® Green QRT-PCR Master Mix Kit (Stratagene, Agilent Technologies Company, Santa Clara, CA, USA). The amplification reactions were conducted under the following conditions: 30 min at 45°C for RT step followed by 10 min at 94°C, continued by 40 cycles of 20 seg at 94°C, 20 seg at 60°C and 30 seg 72°C. The melting curve was performed between 60°C and 90°C to verify the reaction specificity. For each experiment, the amount of RNA-RLuc and RNA-FLuc for each bicistronic RNA was determined (pmoles) based on an individual standard curve constructed using *in vitro* transcribed RNA as a template. Values were corrected considering the amplification efficiency of each set of primers. The RNA-FLuc/RNA-RLuc ratio was used to compare the amount of bicistronic RNA in each transfection assay.

### Immunoblotting

Western blotting was performed after 20 µg of total proteins were resolved on a 12% SDS-polyacrylamide gel and transferred to a nitrocellulose membrane (Bio-Rad). Immunoblotting were performed using mouse anti- GAPDH (1∶5000, Techni-Science), rabbit anti-Actin (1∶5000, Abcam), goat anti-hnRNPA1 (1∶1000, Santa Cruz Biotechnology) and mouse anti-GFP (1∶5000, Roche) antibodies. Horseradish peroxidase (HRP)-conjugated antibodies (Rockland Immunochemicals) were used as secondary antibodies. Signals were detected by chemiluminescence using the Western Lightning Chemiluminescence Reagent kit, as described by the manufacturer (Perkin-Elmer Life Sciences).

### Statistical Analysis

The statistical data analysis and graphics described in the text were done using the GraphPad v5.03 program (La Jolla, CA 92037, USA), while the BioEdit v7.0.9 (Ibis Biosciences, Carlsbad, CA 92008, USA) and the Vector NTI v11 (Invitrogen™ Life Technologies) programs were used for sequence alignments and analysis.

## Results

### The HIV-1 INS-1 Element Inhibits HIV-1 IRES-mediated Translation Initiation *in vitro* and *ex vivo*

**Figure 1:**
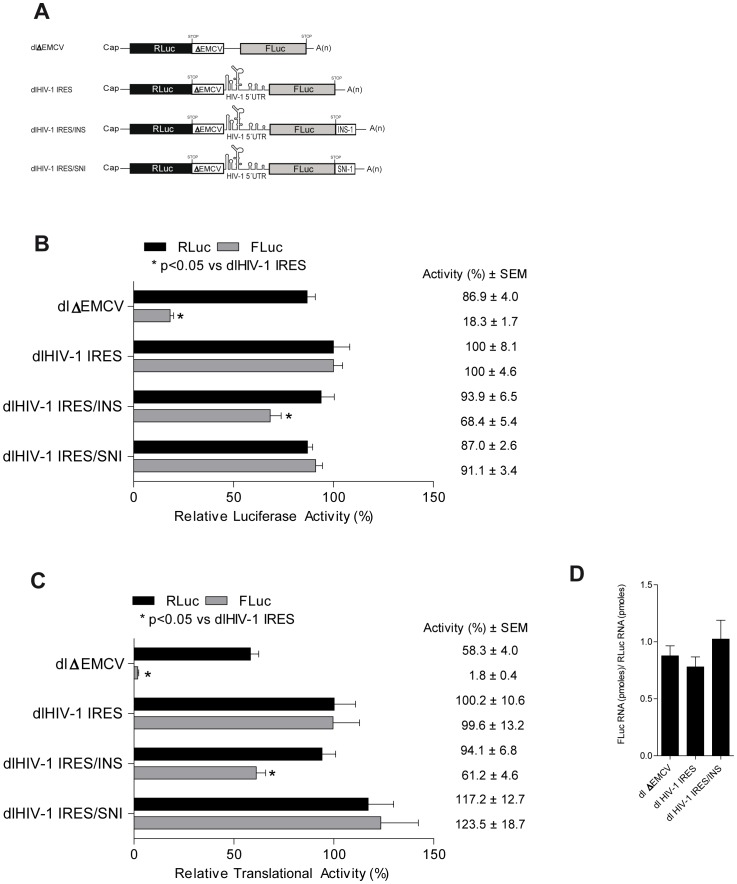
INS-1 sequence negatively impacts on HIV-1 IRES activity. **A)** Schematic representation of the dual luciferase (dl) mRNAs used *in vitro* translation assay. As reporter genes the used bicistronic mRNAs harbor an upstream *Renilla* luciferase (RLuc) and a downstream firefly luciferase (FLuc). INS-1: sense orientation (5′to 3′) and SNI: antisense orientation (3′ to 5′). **B)** Results of *in vitro* translation assay in which luciferase activity values for each cistron corresponds to mean (+/− SEM) of three independent experiments performed each in triplicate. The relative RLuc and FLuc activities for dl HIV-1 IRES were arbitrarily set to 100%. **C)** The luciferase activity was determined 24 h after transfecting HeLa cells with 200 ng of each bicistronic plasmid. The figure shows the values of luciferase activities corresponding to the mean (+/− SEM) of three independent experiments performed each in triplicate. The relative RLuc and FLuc activities for dl HIV-1 IRES were arbitrarily set to 100%. D**)** Total RNA (200 ng) extracted from transfected HeLa cells was used as template in parallel RT-qPCR reactions designed to specifically detect the RLuc or FLuc containing RNAs. The RNA-FLuc concentration (pmoles)/RNA-RLuc (pmoles) ratio was calculated. Values are the means (+/− SEM) from three independent experiments. Statistical analysis was performed by ANOVA test followed by Dunnet multiple comparison. *p<0.05 v/s dlHIV-IRES.

The *gag* coding region inhibits cap-dependent translation from the full-length HIV-1 mRNA [19], [21], [23], [24]. This inhibition is also observed when the *gag* open reading frame (ORF) is in the context of a non-viral heterologous monocistronic mRNA [19], . In HeLa based translational extracts, the inclusion of the *gag*ORF downstream of the HIV-1 5′UTR in the context of a dual luciferase (dl) mRNA, containing an upstream *Renilla* luciferase gene (RLuc) and a downstream firefly luciferase gene (FLuc), inhibits translation of the second cistron [6]. Several reports establish that cap-dependent translation initiation from the HIV-1 mRNA is inhibited by the INS elements [19]–[24]. Based on these observations, we sought to evaluate if the INS-1, cis-acting element, present within the HIV-1 Matrix (p17) Gag coding region, could account for the inhibition of HIV-1 IRES described by Brasey et al. [6]. Following the same experimental approach that was used to establish that INS-1 inhibits cap-dependent translation initiation, the INS-1 was placed after the stop codon of the second cistron in the context of the dl HIV-1 IRES bicistronic mRNA, that harbors the HIV-1 5′UTR within the intercistronic space [6]. Rabbit reticulocyte lysate supplemented with G2/M HeLa extracts (herein referred to as RRL), an *in vitro* translation system known to support HIV-1 IRES activity [11], [34], was programmed with equivalent amounts of capped and polyadenylated dl HIV-1 IRES and dl HIV-1 IRES/INS mRNAs (Fig. 1A). As a control for these series of experiments, the dl HIV-1 IRES/SNI RNA that harbors the inverted INS-1 sequence (SNI), and which is expected to have no impact gene expression [21], [23], [24], [35], was included (Fig. 1A). Results were expressed as relative luciferase activity (RLA), with the mean luciferase activity of the RLuc and FLuc reporters expressed from the dl HIV-1 IRES RNA arbitrarily set to 100% (+/− SEM). Consistent with previous reports [21], [24], [35], SNI did not impact FLuc activity (see dl HIV-1 IRES/SNI in Fig. 1B). When the INS-1 was positioned downstream from the FLuc stop codon, translation from the second cistron was reduced (see dl HIV-1 IRES/INS in Fig. 1B). No effect was observed on the activity of the RLuc reporter encoded by the first cistron, again suggesting that the INS-1 impacts on protein synthesis and not on RNA stability. Thus, similar to what has been described for cap-dependent translation initiation [24], in RRL the INS-1 region negatively impacts on HIV-1 IRES activity.

Next, we evaluated the impact of the INS-1 on HIV-1 IRES activity in cells. For this, plasmids dl ΔEMCV, dl HIV-1 IRES, dl HIV-1 IRES/INS, and dl HIV-1 IRES/SNI (Fig. 1A), were transfected in HeLa cells; cells known to support HIV-1 IRES mediated translation initiation [6], [11], [17]. The RLuc/FLuc bicistronic plasmid dl ΔEMCV, that harbors a defective encephalomyocarditis virus (ΔEMCV) IRES known to inhibit ribosome reinitiation and readthrough, inserted upstream of the FLuc reporter, was used as a negative control [6]. After 24 h, total RNA and proteins were recovered. Proteins were used to measure the luciferase activities. Results were expressed relative to the RLuc or FLuc activity obtained with the control dl HIV-1 IRES vector, which was arbitrarily set to 100% (Fig. 1C). When the INS-1 was appended to the vector after the FLuc stop codon HIV-1 IRES activity was reduced (Fig. 1C). Expression from the first cistron, RLuc, was not affected in dl HIV-1 IRES/INS (Fig. 1C), suggesting that stability of the bicistronic mRNA was not impaired. As before (Fig. 1B), SNI had no effect on FLuc translation (Fig. 1C). To further confirm that RNA stability was not altered by the presence of the INS-1 we quantified the amount of bicistronic mRNA by individually amplifying RLuc or FLuc using a quantitative RT-qPCR assay [34]. For this, total RNA extracted from cells transfected with dl ΔEMCV, dl HIV-1 IRES, and dl HIV-1 IRES/INS was amplified in parallel reactions using primers specifically designed to recognize RLuc or FLuc [34]. The RNA concentration (pmoles) obtained by quantifying the FLuc cistron was divided by the RNA concentration (pmoles) obtained by quantifying RLuc cistron (Fig. 1D). Results show that for the different RNAs the RNA-FLuc/RNA-RLuc ratio was close to 1 (Fig. 1D), indicating that similar amounts of each cistron coding mRNA; dl ΔEMCV (ratio of 0,89), dl HIV-1 IRES (ratio of 0,78), dl HIV-1 IRES/INS (ratio of 1,02). These findings (Fig. 1D), together with the luciferase activity measurements (Fig. 1C) further suggest that the INS-1 negatively impact on protein synthesis and not on RNA stability.

### The HIV-1 INS-1 does not Impair Protein Expression Driven by the HCV IRES in RRL

**Figure 2:**
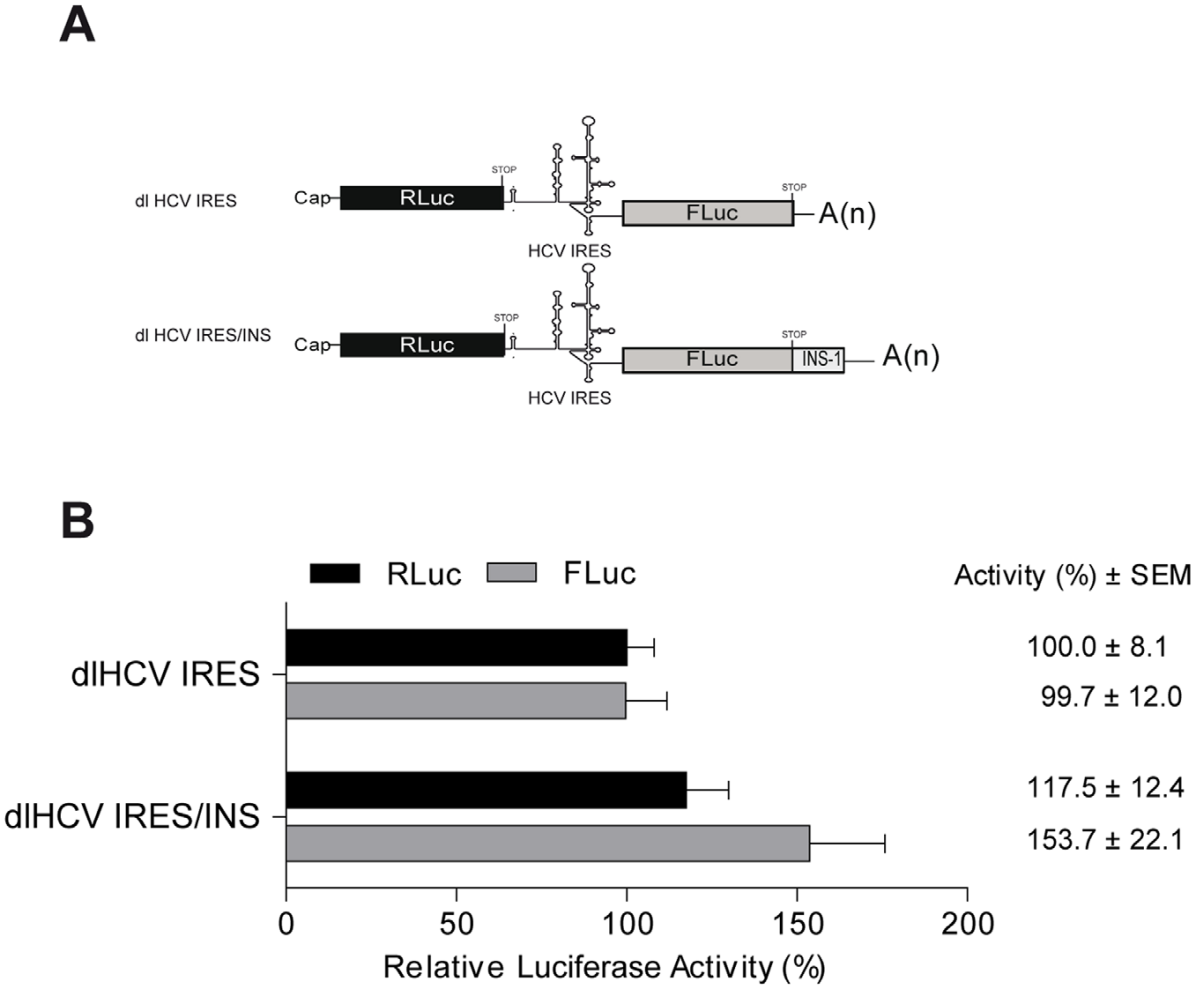
INS-1 sequence does not affect HCV IRES mediated translation. **A**) Schematic representation of the mRNAs used in vitro translation assay [31]. **B**) Results of in vitro translation assay in which luciferase activity values for each cistron are the mean (+/− SEM) of three independent experiments performed each in triplicate. The relative RLuc and FLuc activities for dl HCV IRES were arbitrarily set to 100%.

It was of interest to determine whether the inhibitory effect exerted by the INS-1 could also impact other viral IRESs. The hepatitis C virus (HCV) IRES was selected for these assays as its mechanism of ribosome recruitment is well characterized [3], [36]. As before, the HIV-1 INS-1 was cloned downstream of the stop codon of the FLuc reporter in the context of the previously characterized dl HCV IRES vector [31], generating the dl HCV IRES/INS vector (Fig. 2A). Capped and polyadenylated RNA produced from the dl HCV IRES and dl HCV IRES/INS vectors were *in vitro* translated as described in Materials and Methods. The FLuc and RLuc activities were measured and the results were expressed relative to the RLuc or FLuc activity obtained with the control dl HCV IRES vector, which was arbitrarily set to 100% (Fig. 2B). In sharp contrast to what is seen for the HIV-1 IRES (Fig. 1), *in vitro* INS-1 does not inhibit translation from the HCV IRES. Furthermore, translation initiation was increased in both cistrons suggesting that INS-1 does not destabilize this reporter mRNA.

### The HIV-1 Rev Protein Partially Overcomes INS-1 Induced Inhibition of IRES Activity

**Figure 3:**
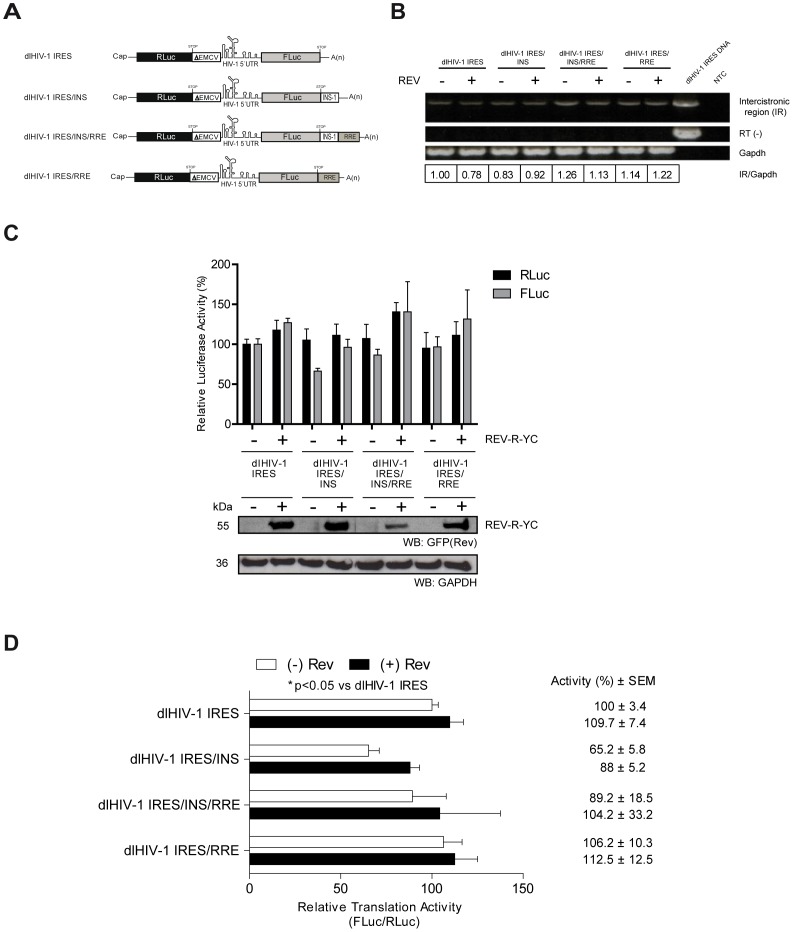
Rev partially rescues the INS-1 inhibitory activity. **A)** Schematic representation of the mRNAs used in *ex vivo* translation assay. HeLa cells were transfected with 250 ng of Rev viral protein (pRev-R-YC; [33]) or pRFP and 200 ng of each bicistronic vector. The luciferase activity was determined 24 h post-transfection. **B)** The transcription of each construct bicistronic was analyzed by RT-PCR from RNA purified from transfected cells. The upper panel shows RT-PCR on intercistronic region mRNA, the medium panel shows the omission of reverse transcriptase RT(−), reaction control (dl HIV-1 IRES DNA) and no template control (NTC), while the lower panel showed the amplicon for the *Gapdh* mRNA. The ratio of IR/Gapdh amplicons is shown. **C)** Relative RLuc and FLuc activities for the different vectors. The RLuc and FLuc activities of the dl HIV-1 IRES in absence of the Rev viral protein were arbitrarily set to 100%. The bottom panel in C shows expression levels of Rev and GAPDH proteins (Western Blot). The recombinant Rev protein was detected using an anti-GFP antibody as indicated in the Materials and Methods section. **D)** The [FLuc/RLuc] ratio used as an index of IRES activity. The figure shows the values of luciferase activities corresponding to the mean (+/− SEM) of three independent experiments.

In the context of monocistronic mRNAs, the inhibitory effect over Gag protein synthesis induced by INS-1 is overcome by the HIV-1 Rev protein [19], [20], [23], [24], [32]. Based on this observation, the effect of Rev on translation of INS-1 containing bicistronic mRNAs was evaluated. As the function of Rev in HIV-1 replication cycle is dependent on its interaction with a highly structured RNA element known as the Rev response element (RRE) [37], two additional vectors: dl HIV-1 IRES/RRE and dl HIV-1 IRES/INS/RRE, harboring the RRE (recovered from clone pNL4.3) after the FLuc stop codon, were designed (Fig. 3A). Plasmids dl HIV-1 IRES, dl HIV-1 IRES/INS, dl HIV-1 IRES/INS/RRE, and dl HIV-1 IRES/RRE (Fig. 3A) were co-transfected into HeLa cells with the pRFP or with the pRev-R-YC plasmid [33]. The pRev-R-YC plasmid expresses the HIV-1 Rev protein in fusion with the monomeric red fluorescent protein fused to the YC domain (amino acids 155–238) of the yellow fluorescent protein [33]. After 24 h, total RNA and proteins were recovered. RNA was used to evaluate whether bicistronic mRNA was indeed expressed in our experimental setting (Fig. 3B), as previously described [11], [34]. The expected amplicon was identified confirming the presence of the full-length bicistronic mRNA in all cases (Fig. 3B). No product was observed when the PCR reaction was conducted without a previous step of reverse transcription, confirming the absence of DNA contamination in the RNA preparation (Fig. 3B). The ratio of intercistronic region (IR) to Gapdh amplicons are shown (Fig. 3B). Proteins were used to determine the FLuc and RLuc activities and to confirm, by Western Blot analysis, the presence of the pRev-R-YC protein. As shown in Figure 3C, the pRev-R-YC protein was readily detected using a commercial anti-GFP antibody, which detects the YC domain of the protein. Luciferase activities were expressed relative to the RLuc or FLuc activity obtained with the control dl HIV-1 IRES vector, which was arbitrarily set to 100% (Fig. 3C). The presence of the RRE element had no impact on the activity of the HIV-1 IRES (compare dl HIV IRES and dl HIV-1 IRES/RRE). Addition of the pRev-R-YC protein increased gene expression from all constructs (compare (−) and (+) pRev-R-YC lanes in Fig. 3C). The presence of Rev is capable of partially restoring gene expression from INS-1 regulated cistrons (Fig. 3C). To better evaluate the impact of the pRev-R-YC protein on IRES driven translation initiation, we decided to use the FLuc/RLuc ratio as the readout of IRES activity. Results expressed as relative translation activity (RTA), with the mean translation efficiency of the dl HIV-1 IRES vector arbitrarily set to 100% (+/− SEM), are shown in Figure 3D. Results confirm that the presence of the RRE element has no direct impact on the activity of the HIV-1 IRES (Fig. 3D, compare dl HIV IRES and dl HIV-1 IRES/RRE). The presence of pRev-R-YC protein, on the other hand, impacts the activity of the HIV-1 IRES even in the absence of the RRE sequence (Fig. 3D, compare dl HIV-1 IRES/INS and dl HIV-1 IRES/INS/RRE). These last observations suggest that the effect of pRev-R-YC on HIV-1 IRES mediated translation is independent of the RRE and suggest that the presence of the viral protein REV stimulates the activity of the HIV-1 IRES when in presence of the INS-1.

### Heterogeneous Nuclear Ribonucleoprotein A1 Modulated HIV-1 IRES-activity

**Figure 4:**
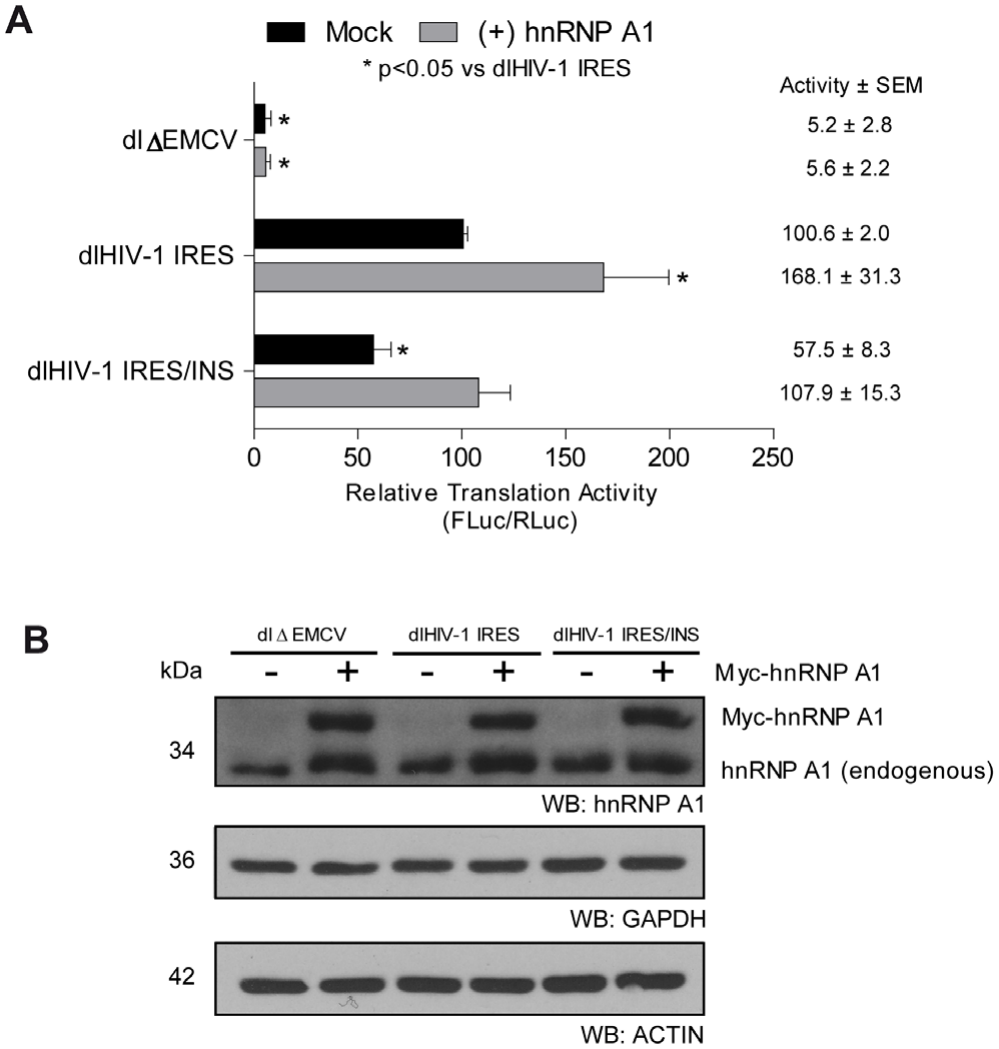
hnRNPA1 rescues the INS-1 inhibitory activity. **A)** HeLa cells were transfected with hnRNPA1 expressing vector (1 µg) or pcDNA3.1 (1 µg), and each bicistronic construct (1 µg). Luciferase activity was determined 24 h post-transfection. The [FLuc/RLuc] ratio was used as an index of IRES activity. The value for the [FLuc/RLuc] ratio of the dl HIV-1 IRES vector was arbitrarily set to 100%. **B)** Proteins extracts from the cells were used to evaluate the expression levels of hnRNPA1, GAPDH and Actin by Western Blotting. Statistical analysis was done by ANOVA test followed by Dunnet multiple comparison, *p<0.05 v/s dlHIV-IRES.

Several events in the HIV-1 replication cycle are orchestrated by a diversity of viral and host proteins that interact with each other and with the viral RNA to form HIV-1 ribonucleoprotein (RNP) complexes [38]. Recent studies from our laboratories suggest that the heterogeneous nuclear ribonucleoprotein A1 (hnRNP A1), a protein known to associate with the viral RNA in the nucleus as part of the HIV-1 RNP, plays a role as a positive modulator of HIV-1 IRES activity [17]. Interestingly, hnRNP A1 binds to the full length HIV-1 RNA [39], [40], and more specifically to the INS-1 element [28], [39], [40]. These observations prompted us to evaluate the possible role of hnRNP A1 on the function of INS-1. Plasmids dl ΔEMCV, dl HIV-1 IRES, dl HIV-1 IRES/INS, were co-transfected in HeLa cells with plasmid pcDNA3.1 or with a previously characterized plasmid expressing hnRNPA1 [17]. After 24 h, total proteins were recovered and the FLuc and RLuc activities were measured as described in Materials and Methods. The FLuc/RLuc ratio was used as the readout of IRES activity expressed as RTA, with the mean translation efficiency of the reference IRES (dl HIV-1 IRES vector) arbitrarily set to 100% (+/− SEM) (Fig. 4A). The expression of the transfected DNA encoding the myc-tagged hnRNPA1 protein was confirmed by Western blotting (Fig. 4B, upper panel). In concordance with the above presented data, IRES activity in the dl HIV-1 IRES/INS vector was reduced by more than 43% when compared to the reference vector (Fig. 4A, compare black bars). In agreement with the report of Monette et al. (2009), over expression of hnRNP A1 stimulated translational activity from the dl HIV-1 IRES RNA (Fig. 4A). However, in construct dl HIV-1 IRES/INS, the previously described stimulation of IRES activity was not observed. Interestingly, in the presence of over expressed hnRNP A1 the inhibitory effect exerted by the INS-1 element was repressed and HIV-1 IRES activity was recovered (Fig. 4A). This observation suggests that hnRNP A1 modulates the function of *cis*-acting repressing elements.

## Discussion

Several cis-acting RNA elements located within the HIV-1 mRNA coding region [19]–[24], [41], RNA structural elements in the HIV-1 5′leader [7], [42]–[48], as well as viral [49] and cellular proteins (reviewed in [50]) are known to regulate HIV-1 gene expression. A number of studies have documented that the complete *gag*ORF inhibits Gag protein expression by reducing RNA stability and by targeting translation initiation [6], [19]–[24], [41]. In addition to the full length *gag*ORF, RNA inhibitory sequences or INS present within the *gag*ORF are sufficient to inhibit cap-dependent translation initiation. In this study, we evaluated the impact of the INS-1 on the activity of the HIV-1 IRES, showing that HIV-1 IRES mediated translation initiation is inhibited by INS-1. The presence of the INS-1 did not impact on RLuc activity, encoded by the first cistron suggesting that RNA stability was not altered (Fig. 1). Together, these observations indicate that the INS-1 inhibits HIV-1 IRES activity. Results also confirm that translation inhibition exerted by the INS-1 acts in an orientation-dependent manner (Fig. 1). Reports suggest that the INS-1 acts as a suppressor of cap-dependent translation initiation [19], [24]. The presented data indicate that the INS-1 also affects IRES-mediated translation initiation (Fig. 1), however this inhibitory effect is not general to all viral IRESes as the INS-1 did not inhibit HCV IRES activity (Fig. 2).

The molecular mechanism by which the INS-1 negatively impacts on gene expression remains unclear [19], [24], [32]. Several studies however suggest that the inhibitory function of the INS-1 is mediated by viral and cellular proteins [19], [20], [23]–[27], [39]. When assessed in the context of a monocistronic [19], [20], [23], [24] or a bicistronic (Fig. 3) mRNA, the inhibitory activity of the INS-1 can be overcome by the viral regulatory factor Rev. The mechanism by which Rev relieves the INS-1 inhibitory activity remains unclear as the increase of reporter protein expression was independent of the presence of the primary Rev binding site, RRE (Fig. 3 and [24], [51]). These findings seem to contradict several other reports showing that the function of Rev is associated to its ability to bind viral RNA [20], [35], [37], [52], [53]. Nonetheless, Rev also binds to the 5′UTR of the unspliced HIV-1 mRNA, but with a much lower affinity, than to the RRE [54]. Furthermore, Rev stimulates translation from the HIV-1 5′UTR in an RRE independent fashion [51]. Thus, results suggest that it is the interaction of Rev with this secondary binding site within the HIV-1 5′UTR that relieves the inhibition imposed by the INS-1 through a yet uncharacterized mechanism (Fig. 3).

A number of cellular proteins known to bind the INS sequences alter HIV-1 Gag protein synthesis [25], [26], [28]. In this context, hnRNP A1 represented a suitable candidate protein to evaluate as it directly binds to the INS-1 element [28], [39], [40] and is known to stimulate HIV-1 IRES [17]. Stimulation of HIV-1 IRES activity by hnRNP A1 is independent from other viral proteins yet relies on its ability to bind the viral mRNA [17]. Additionally hnRNP A1 is known to be involved in the replication of several viruses including the HCV, dengue virus, the human T-cell leukemia virus type I, and human papillomavirus type 16 among others, through an interaction with regulatory regions present within the viral mRNAs [55]–[59]. When overexpressed, hnRNP A1 partially relieved the inhibitory effect of the INS-1 element on HIV-1 IRES activity (Fig. 4), further suggesting the involvement of cellular proteins in the modulation of HIV-1 IRES activity [11], [16]–[18]. Translation of the HIV-1 mRNA is known to be regulated by several other cellular proteins for example, the export receptor Tap/NXF1, RNA helicase A, splicing factor 9G8, and Src-associated in mitosis 68 are examples of nucleocytoplasmic shuttling proteins that promote viral mRNA translation, while hnRNP E1 negatively affects HIV-1 translation (reviewed in [50]). Several cellular proteins have been proposed to directly modulate HIV-1 IRES activity, for example the human embryonic-lethal abnormal vision (ELAV)-like protein HuR inhibits HIV-1 IRES activity [18], while the eukaryotic translation initiation factor 5A, the human Rev-interacting protein, and the DEAD (Asp-Glu-Ala-Asp) box polypeptide 3, and hnRNPA1 stimulate translation from the IRES [16], [17]. Together these findings stress the role of cellular proteins as modulators of HIV-1 IRES activity.

Recent reports question the role of RNA structure on translation driven by the HIV-1 5′UTR activity yet underscore the possible function of cellular proteins complexes in HIV-1 IRES mediated protein synthesis [11], [60]. Mutations designed to alter the structure of the HIV-1 5′UTR do not impact on HIV-1 IRES activity [11]. However, HIV-1 IRES activity is poor in G1 phase of the cell cycle, suggesting that structure alone does not account for IRES activity [11]. Interestingly, the protection pattern of the HIV-1 5′UTR varies sharply in the presence of protein extracts that favor IRES activity when compared to those that do not [11]. Together these observations suggest that different RNPs would assemble on to the viral RNA generating different protection patters and modulating IRES function. In this context, RNA elements such as IRENE [7], or the INS (data herein) would act in concert with a specific set of proteins in the overall fine tuning of HIV-1 translational activity. This mode of action would explain why HCV IRES translational activity is not inhibited by the INS-1 (Fig. 2). Activity of the HCV IRES is exclusively dependent on its RNA structure and formation of the 40S/HCV IRES complex occurs without the requirement of initiation factors or cellular proteins [61]–[64]. If the INS-1 or the INS-1/protein complex, that in the herein used vectors are downstream of the second cistron, do not disrupt or blocks the HCV IRES structure then they should not be expected to inhibit its activity either.

In cells, an important step in controlling protein synthesis is translation initiation, and hence this process is a target of diverse signaling pathways [1], [2]. It is therefore plausible that several mechanisms which involve *cis*-acting RNA elements, viral proteins, and cellular proteins, act in concert to repress or activate both cap- and IRES-dependent initiation of HIV-1 mRNA. Further experiments are required to identify other cellular proteins members of the protein complex responsible of mediating the INS-1 inhibition of Gag protein synthesis.
